# Pyroptosis-Related Patterns Predict Tumor Immune Landscape and Immunotherapy Response in Bladder Cancer

**DOI:** 10.3389/fmolb.2022.815290

**Published:** 2022-04-26

**Authors:** Yilin Yan, Xiangqian Cao, Zeyi Wang, Zhengnan Huang, Jinming Cai, Pengfei Tang, Chenkai Yang, Fang Zhang, Shujie Xia, Bing Shen

**Affiliations:** ^1^ Department of Urology, Shanghai General Hospital, Shanghai Jiaotong University School of Medicine, Shanghai, China; ^2^ Department of Urology, Shanghai General Hospital Affiliated to Nanjing Medical University, Shanghai, China; ^3^ Institute of Urology, Shanghai Jiao Tong University, Shanghai, China

**Keywords:** bladder cancer, pyroptosis, score, immunotherapy, microenvironment

## Abstract

**Background:** Bladder cancer (BC) is a leading cause of death from malignancy, with significant heterogeneity in the immunotherapeutic responsiveness of advanced status. Pyroptosis, a newly discovered inflammatory programmed cell death, is confirmed to play an indispensable role in tumorigenesis and anti-tumor activity. However, the effect of pyroptosis on the tumor-immune landscape remodeling and immunotherapy in BC remains elusive.

**Methods:** We comprehensively evaluated the mRNA expression and genomic alterations of 33 pyroptosis-related genes (PRGs) in BC and evaluated the patterns of pyroptosis in publicly available BC datasets. An unsupervised clustering method was used to classify patients into distinct patterns. Then, we established a pyroptosis-related signature score (PS-score) model to quantify the pyroptosis-related patterns of individual BC patients using principal component analysis. Furthermore, we correlated the patterns with the immune landscape and response efficacy of immunotherapy.

**Results:** Two pyroptosis-related patterns were identified in BC, and distinct patterns showed various immune characteristics. Patterns with a high expression level of PRGs exhibited a survival advantage and showed higher infiltration of cytotoxic lymphocytes. Tumors with a low PS-score were characterized by high tumor-infiltrating lymphocytes and considered “hot.” Further analysis revealed that the PS-score was an independent prognostic factor and could predict the response to immunotherapy for patients with advanced BC. We found a significant positive association between AHNAK2, AHNAK nucleoprotein 2, expression, and PS-score. Functional assays showed that AHNAK2 knockdown was correlated with attenuated invasive ability.

**Conclusion:** This work comprehensively demonstrated the potential function of pyroptosis-related patterns in the bladder tumor-immune landscape and identified their therapeutic liability in immunotherapy. Our study enhanced our understanding of the immune landscape and provided a new approach toward more effective immunotherapy strategies.

## Introduction

Bladder cancer (BC) remains one of the most prevalent tumors in the urinary system and is characterized by high morbidity and mortality rates (Siegel et al., 2021). BC can be further divided into two subtypes: muscle-invasive bladder cancer (MIBC) and non-muscle-invasive bladder cancer (NMIBC) (Alifrangis et al., 2019). Despite well-established surgical and chemotherapy options in the past decades, recurrence and mortality rates are still significant because of high-level genomic instability and heterogeneity. Although immunotherapy, such as PD ligand 1 (PD-L1)/programmed cell death protein 1 (PD-1) blockade therapy, contributes to curative effectiveness in advanced BC, a low response rate still limits the clinical benefit (Ott et al., 2020; Patel et al., 2020). Consequently, an investigation of prognostic biomarkers for the development of novel therapeutic strategies is urgently needed. Several reports have suggested that the tumor immune microenvironment (TIME) plays a significant role in the response of immunotherapy, which relies on the amounts of tumor-infiltrating lymphocytes (TILs) (Pitt et al., 2016; Zou 2020). Understanding the mechanisms of TIME-modulating immunotherapy response is vital for immunotherapeutic strategies.

Pyroptosis is a novel form of proinflammatory cell death and is characterized by pore formation on the cellular membrane and cellular swelling, followed by releasing inflammatory cytokines such as interleukin (IL)-1β and IL-18 executed by the gasdermin protein family (Shi et al., 2017). In terms of mechanism, pyroptosis relies on the cleavage of the canonical (caspase-1) and non-canonical inflammasome. Activated by inflammasomes, caspase-1 can accelerate the cleavage of gasdermin D (GSDMD) and secretion of pro-inflammatory cytokines (Kang et al., 2018). Other gasdermin molecules, particularly gasdermin E (GSDME), can be cleaved by caspase-3 to induce pyroptosis (Liu et al., 2020). Pyroptosis has been extensively studied in tumor development and cancer immunotherapy in recent years. Previous studies have shown that GSDMD could facilitate the capability of CD8^+^ T cells to kill tumor cells (Xi et al., 2019). Granzyme A (GzmA) was reported to trigger tumor clearance by recruiting NK and CD8^+^ T cells and induce pyroptosis via cleavage of GSDMB (Zhou et al., 2020). GSDME could turn a “cold” tumor into a “hot” status that the immune system cannot recognize when it is activated. Another recent study demonstrated that cytotoxic T cells could promote the cleavage of GSDME to induce cell death by releasing the granzyme B (Zhang et al., 2020).

The aforementioned studies involved only several pyroptosis-related molecules. However, the modulation of the immune response is a complex network, which involves the mutual regulation of many signaling pathways. Recently, several studies have identified a novel pyroptosis-related signature for the prognosis of cancer, such as ovarian cancer and melanoma (Ju et al., 2021; Ye et al., 2021). However, comprehensive studies on the correlation between TIME and pyroptosis-related genes remain poorly explored. Therefore, the extensive investigation of the tumor immune microenvironment mediated by pyroptosis-related molecules will profoundly enhance our understanding of their immunoregulatory function.

Herein, we explored gene expression heterogeneity and genomic alterations and evaluated the patterns of pyroptosis in publicly available BC datasets. Surprisingly, we identified two distinct pyroptosis-related patterns and were surprised to find that they had distinct prognoses and immune characteristics of the microenvironment, indicating the effect of pyroptosis on the formation of the tumor immune microenvironment. We then established a scoring system, pyroptosis-related signature score (PS-score), to quantify the pyroptosis-related patterns of individual BC patients. Finally, we demonstrated the applicability of the PS-score and confirmed its prognostic and therapeutic value in immunotherapy for BC patients.

## Materials and Methods

### Data Collection and Processing

The bladder cancer transcriptome, genetic mutations, and complete clinical data were procured from The Cancer Genome Atlas (TCGA) database (https://portal.gdc.cancer.gov/). The gene expression and clinical information of external validation cohorts were obtained from the GEO database (https://www.ncbi.nlm.nih.gov/geo/, GSE32894 and GSE48075). Bladder cancer patients treated with immunotherapy (atezolizumab) were from IMvigor210 (http://research-pub.Gene.com/imvigor210corebiologies/) (Mariathasan et al., 2018). Patients without prognostic data were removed from further analysis. Immunohistochemical (IHC) images of human bladder samples were obtained from HPA (http://www.proteinatlas.org) (Uhlen et al., 2017). In this research, the level of AHNAK2 protein expression between the urothelium and bladder tumor tissues was visualized by HPA.

### Clustering Pattern of Pyroptosis-Related Genes

Thirty-three pyroptosis-related molecules were retrieved from the previous literature (Man and Kanneganti 2015; Karki and Kanneganti 2019; Xia et al., 2019). An unsupervised clustering analysis (K-means) was applied to determine the optimal clustering number of the expression level of the aforementioned 33 pyroptosis-related molecules with the R package “ConsensusClusterPlus” (Wilkerson and Hayes 2010). The number of clusters (k) was selected based on the Bayesian information criterion.

### Estimation of Immune Cell Infiltration

The gene expression data from TCGA database were used to estimate the infiltration level of immune populations by CIBERSORT, TIMER, MCPCounter, and the ssGSEA algorithm. CIBERSORT was used to calculate 22 types of immune cells in bladder cancer patients (Newman et al., 2015). TIMER (https://cistrome.shinyapps.io/timer/) is a method that could quantify the relative proportion of six immune cell types (Li et al., 2020). The single-sample Gene Set Enrichment Analysis (ssGSEA) was performed using the GSVA Bioconductor package (Hänzelmann et al., 2013). The estimation of immune cell fractions was determined through gene expression level using MCPCounter deconvolution methods (Becht et al., 2016).

### Construction and Validation of Pyroptosis Characteristic Signatures

To quantify the pyroptosis characteristic pattern of individual BC patients, we firstly identified DEGs with an absolute value of log2FC ≥ 1 and FDR <0.05 among different patterns with the “limma” package (Ritchie et al., 2015). Then, a univariate Cox regression analysis was applied to assess the prognosis value and 142 prognostic genes were extracted for further principal component analysis (PCA) (Ringnér 2008). Finally, we constructed a scoring system with principal components 1 (PC1) and 2 (PC2) as the final score. The pyroptosis-related signature score (PS-score) for each patient was determined by summing the level of PC1 with its corresponding PC2 value.

### Pathway Enrichment Analysis

DEGs between distinct clusters in TCGA datasets were obtained, followed by carrying out a functional enrichment analysis with Metascape (Zhou et al., 2019). |log2FC| ≥ 1 and a false discovery rate (FDR) < 0.05 were considered as statistically significant. Enrichment of KEGG and other biological processes to obtain the signaling pathway variation score was conducted by the “GSVA” R package, a non-parametric and unsupervised method. We refer to the study of Mariathasan to study the correlation between the PS-score and relevant biological processes (Mariathasan et al., 2018), including immune checkpoint, effector CD8 T-cell signature, antigen-processing machinery (APM), Wnt targets, mismatch repair, DNA replication, and epithelial–mesenchymal transition markers. To identify biological signaling pathways that were significantly alerted between distinct the PS-score, the gene set enrichment analysis (GSEA) was conducted with the Java GSEA software and the gene sets from the database of Kyoto Encyclopedia of Genes and Genomes (KEGG) were selected for reference (Subramanian et al., 2005).

### Correlation of Pyroptosis-Score and Immunotherapy Response

The IMvigor 210 immunotherapeutic cohorts evaluated the anti-PD-L1 antibody (pembrolizumab) efficacy in patients with advanced urothelial cancer (Mariathasan et al., 2018). Transcriptome data and clinical information were downloaded from http://research-pub.Gene.com/imvigor210corebiologies. Patients with partial response (PR) or complete response (CR) were considered responders, whereas non-responders were defined as having progressive disease (PD) or stable disease (SD). The PS-score was then applied to evaluate the prognostic capacity in this cohort.

### Construction and Evaluation of the Nomogram

An established nomogram was constructed to calculate the score for the patients to evaluate the survival probability as a single numerical value. The nomogram construction was performed using the “rms” package (Feng et al., 2017). Apart from this, the model calibration was evaluated by using calibration plots. A decision curve analysis (DCA) was conducted to evaluate the clinical usefulness of the PS-score (Van Calster et al., 2018).

### Transient Transfections

For transient transfection, the control siRNA and AHNAK2 siRNA (GenePharma, Shanghai, China) were synthesized. The transient transfection of siRNA was achieved by the transfection of siRNA oligos using Lipofectamine^®^ 3000 Reagent (Invitrogen) following the manufacturer’s instructions (Yan et al., 2020a). The specific siRNA sequence was as follows: AHNAK2 siRNA-1 sequence 5′- CCA​AGU​GGA​UGU​GAA​ACU​UTT-3′; AHNAK2 siRNA-2 sequence 5′- GCC​CUG​AAA​UAG​ACA​UCA​ATT-3′; siNC (noncoding control): 5′- UUC​UCC​GAA​CGU​GUC​ACG​U -3′.

### RNA Extraction and RT-qPCR

The total RNA was extracted using TRIzol Reagent (Invitrogen) and then reverse-transcribed by PrimeScript™ RT Master Mix (TaKaRa) following the manufacturer’s instructions (Yan et al., 2021). An RT-qPCR was then performed on the StepOnePlus machine utilizing TB Green Premix Ex Taq II (TaKaRa). The primers were as follows: AHNAK2 forward 5′- GTG​CAG​AAA​CGG​AAG​ATG​ACC-3′, reverse 5′- GCC​TCA​GTC​GTG​TAT​TCG​TAG​A-3′; GAPDH forward 5′- CTG​GGC​TAC​ACT​GAG​CAC​C-3′, reverse 5′- AAG​TGG​TCG​TTG​AGG​GCA​ATG-3′.

### Transwell Invasion Assay

The transwell assay for invasion was performed in transwell inserts with an 8.0-μm-pore polycarbonate membrane as described previously (Yan et al., 2020a). In brief, when the transwell chamber was coated with Matrigel, 10^5^ cells were seeded on the upper chamber and incubated for 8 h. After this, the cells were fixed with 4% formaldehyde, stained with 0.5% crystal violet, and visualized with light microscopy.

### Statistical Analysis

The Wilcoxon test was performed to compare the gene expression level among distinct subgroups. Normal variables were assessed with the unpaired student’s t-test, while non-normal variables were analyzed by the Mann–hitney *U* test. Survival curves were generated with the Kaplan–Meier method, and the log-rank test was used to determine statistically significant differences. The “Surv-cut point” in the “Survminer” R package was utilized to evaluate the best cut-off value of each subgroup. Spearman’s correlation analysis was used to calculate the correlation coefficient. Results of the univariate and multivariate Cox regression analyses were generated using the “forestplot” R package. In this research, we considered *p* < 0.05 as statistical significance.

## Results

### Landscape of Genetic and Transcriptional Alterations of Pyroptosis-Related Genes in Bladder Cancer

**FIGURE 1:**
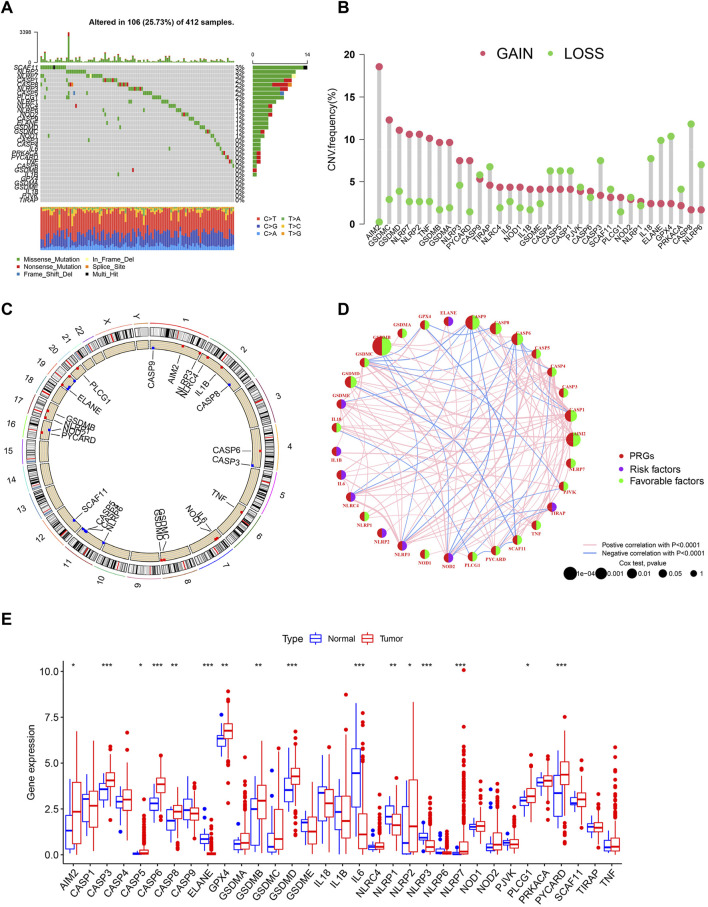
Landscape of transcriptional and genetic variations of PRGs in BC. **(A)** The distribution of somatic mutation in BC patients from TCGA cohort. The *y*-axis represents the gene and the *x*-axis represents patients. **(B)** Dumbbell plot showing the CNV frequency of PRGs in BC. The amplification (red) or deletion (green) frequencies are shown. **(C)** The location of CNV alterations of PRGs on chromosomes in TCGA cohort. CNV: copy number variations. **(D)** The interactions among PRGs in BC. A positive correlation is labeled with red and a negative correlation is marked with blue. **(E)** Expression of PRGs in normal urothelium and tumor tissues. *p < 0.05, **p < 0.01, ***p < 0.001.

According to the previous reports, we identified 33 molecules that modulate pyroptosis. To determine the genetic alterations of pyroptosis-related genes (PRGs) in BC, we assessed the prevalence of somatic mutations of these genes (Figure 1A). The overall average mutation frequency was high, with 106 (25.73%) of 412 samples having mutations. Among them, the mutation frequencies of SCAF11 (3%), NLRP2 (3%), and NLRP7 (3%) were the highest, followed by CASP1 (2%), CASP5 (2%), CASP8 (2%), and NLRP3 (2%). We then examined the incidence of copy number variations and found that AIM2, GASDMC, and GASDMD had a widespread frequency of copy number variation (CNV) gain (Figure 1B). Figure 1C shows the location of CNV alterations of these genes on the chromosomes. The comprehensive picture of PRG interactions and their prognostic value for BC was explored with a network (Figure 1D). A simple forest plot of the hazard ratio for each gene of the 33 PRGs is shown in Supplementary Figure S1. We identified that the expression levels of these genes had a high expression correlation with each other. Moreover, many PRGs were closely associated with the prognosis in BC patients. The expression levels of PRGs in tumor tissues and normal urothelium were also investigated, showing that 16 out of 33 genes were differentially expressed (Figure 1E). More specifically, the expression of AIM3, CASP3, CASP5, CASP6, CASP8, GPX4, GSDMB, GSDMD, NLRP2, NLRP7, PLCG1, and PYCARD was increased, while the expression of ELANE, IL6, NLRP1, and NLRP3 was decreased in bladder cancer compared with normal tissues. Taken together, these results indicated a genetic and expression landscape of PRGs, indicating that the imbalance of these genes had a potential role in the development of BC.

### Identification of Pyroptosis Subtypes and Biological Characteristics of Each Subtype in Bladder Cancer

**FIGURE 2:**
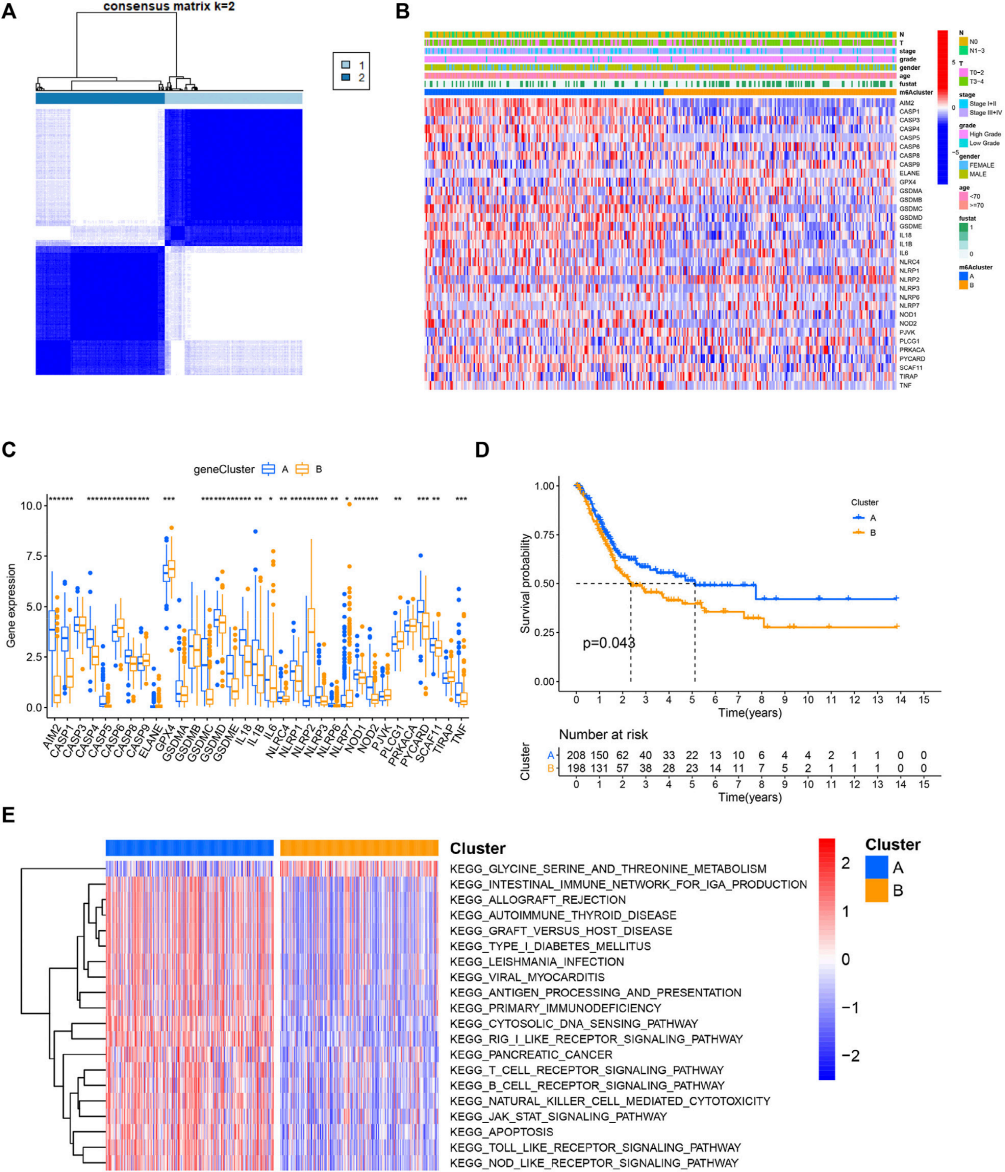
Identification of pyroptosis subtypes and biological characteristics of each subtype in BC. **(A)** Patients with BC are grouped into two clusters according to the consensus clustering matrix (k = 2). **(B,C)** Heatmap and boxplot showing the expression level of PRGs and the clinicopathological characteristics of the two clusters classified by PRGs. **(D)** Kaplan–Meier curves compare prognosis between the two subtypes. **(E)** The heatmap depicting the activation status of biological signaling evaluated by GSVA in distinct clusters. Red: activated pathways and blue: inhibited pathways. *p < 0.05, **p < 0.01, ***p < 0.001.

Based on the gene expression of 33 PRGs, we applied the consensus clustering method to classify patients with different RNA modification patterns. We ultimately uncovered two subtypes (identified as pyroptosis-related clusters A–B), including 210 cases in cluster A and 198 cases in cluster B (Figure 2A). A consensus CDF plot and consensus index for k = 2 to 9 are represented Supplementary Figure S2.

The gene expression profile of the 33 genes and clinical features were presented in a heatmap. Several clinicopathological variables, such as lymph node metastasis and a higher malignant stage were enriched in patients classified in cluster B (Figure 2B, Supplementary Figure S3). Moreover, the majority of these genes were differentially expressed between the two clusters (Figure 2C). In the prognostic analysis, pyroptosis-related cluster A had a particularly favorable survival advantage (Figure 2D). To investigate the biological significance between the two clusters, GSVA was then performed. Surprisingly, cluster A was markedly enriched in pathways associated with apoptosis and immune activation, including antigen processing and presentation, T cell receptor signaling pathway, B cell receptor signaling pathway, and natural killer cell-mediated cytotoxicity (Figure 2E).

### The Immune and Genetic Landscape in Distinct Pyroptosis Clusters

**FIGURE 3:**
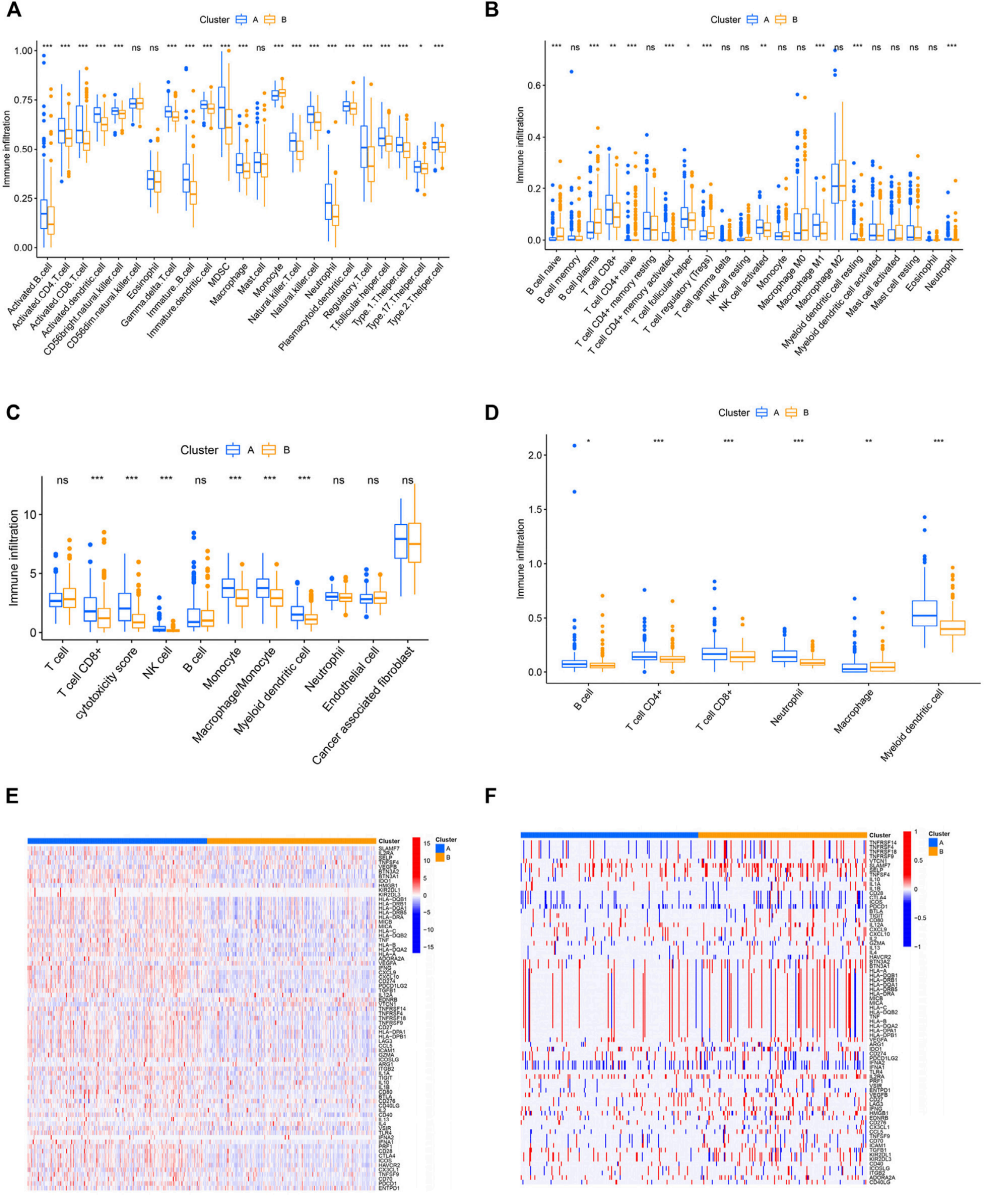
Immune and genetic landscapes in distinct pyroptosis clusters. **(A–D)** Difference of immune cell infiltration estimated by ssGSEA, cibersort, MCPCOUNTER, and TIMER. **(E,F)** Heatmap showing expression and somatic mutations of 75 immunomodulator genes between the two clusters. *p < 0.05, **p < 0.01, ***p < 0.001.

The aforementioned results indicated that the pyroptosis-related cluster was associated with immune-associated signaling pathways. Thus, we next analyzed tumor-infiltrating immune cell (TIIC) proportions by using the algorithms of ssGSEA, cibersort, TIMER, and MCP-counter, widely recognized methods for estimating immune cell infiltration. Two distinct immune infiltrate patterns were discovered in the two clusters. We found that activated tumor-infiltrating lymphocytes were enriched in cluster A, especially activated CD4^+^ cells, CD8^+^ T cells, and NK cells (Figures 3A–D). Immunomodulators (IMs) are essential for cancer immunotherapy being explored in tumor interventions (Yang 2015; Tang et al., 2018). To conduct this study, an investigation of their expression profiles in distinct clusters is urgently required. We thus explored the expression level and CNV alterations among the pyroptosis-related patterns and found that the gene expression and CNVs of IMs varied across distinct subtypes (Figures 3E,F). The two clusters exhibited both frequent amplification and deletion of these genes. Differential somatic mutations were detected within the two patterns and compared with the “maftools” package (Supplementary Figures S4A, B).

### Construction and Validation of Pyroptosis Characteristic Signatures

**FIGURE 4:**
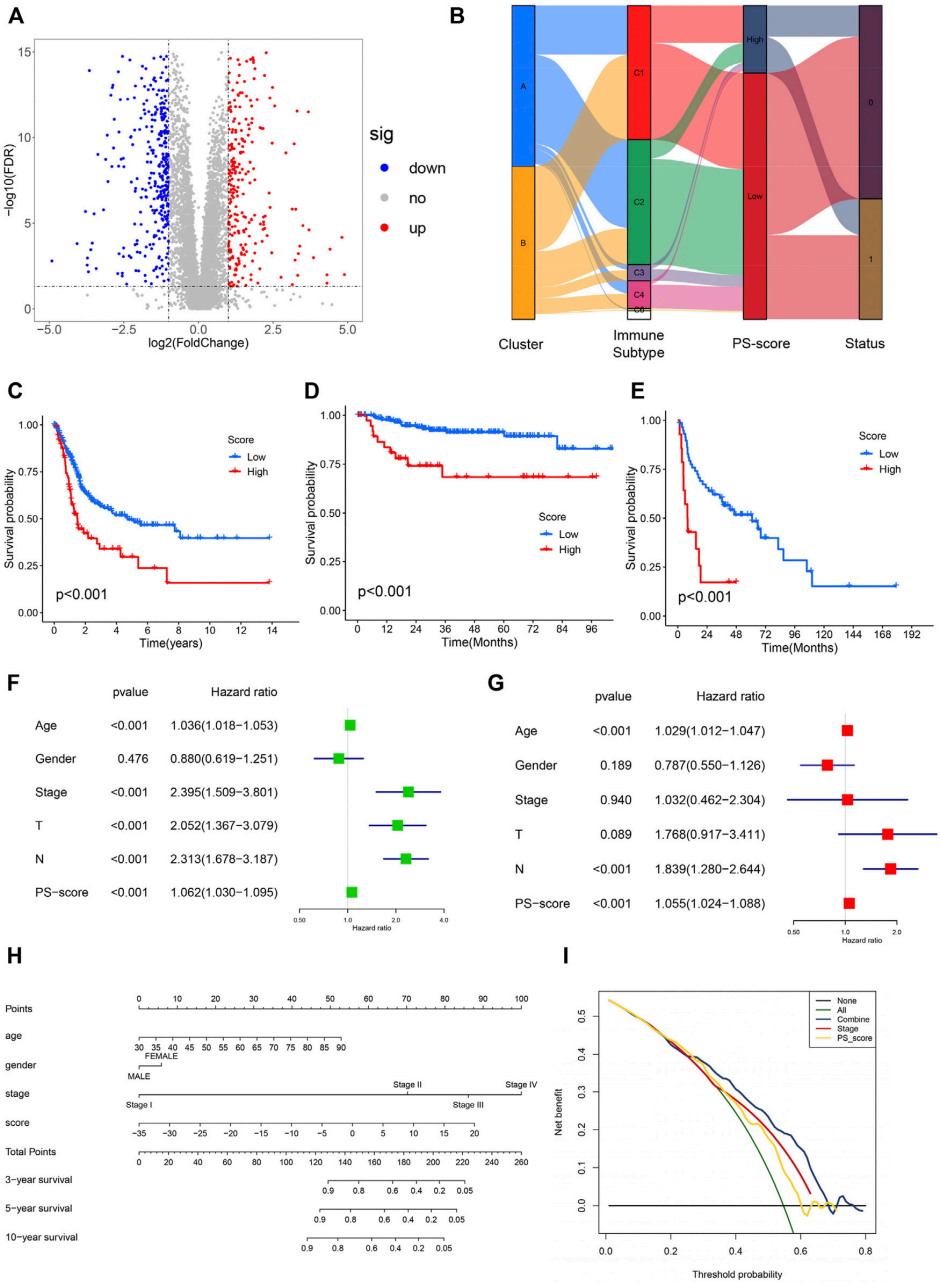
Construction and validation of pyroptosis-characteristic signature. **(A)** Volcano plot presenting the differentially expressed genes (DEGs) between two pyroptosis-related groups. **(B)** Sankey plot of distribution in subgroups with distinct pyroptosis-related clusters, PS-score, and survival status. **(C–E)** Kaplan–Meier survival curves for patients of TCGA, GSE32894, and GSE48075 cohorts to estimate the overall survival based on the PS-score. **(F,G)** Results of univariate and multivariate regression analyses for the PS-score are presented via the forest plot. **(H)** Nomogram for survival prediction in patients with bladder cancer to predict the survival rates of BC patients at 3, 5, and 10 years. **(I)** Decision curve analysis (DCA) of OS for the nomogram model. The *y*-axis represents the net benefit, and the *x*-axis represents the threshold probability. The green curve represents that all patients were considered with poor overall survival. The blue curve shows that the combined model is optimal for decision making for maximal net benefit and adds more benefit than using the single stage and PS-score to predict the overall survival of BC patients.

To further accurately evaluate the functional role of the two subtypes, we identified 657 pyroptosis-related differentially expressed genes (|log2FC| ≥ 1 and FDR <0.05) associated with the two patterns (Figure 4A). Next, the univariate analysis was conducted to assess the prognostic capacity and 142 genes were extracted for the PCA to establish a system that could quantify individual BC patients, namely the pyroptosis-related signature score (PS-score). An alluvial diagram was conducted to visualize the alterations in the attributes of individual BC patients (Figure 4B). Comparing PS score levels between the two clusters, it was found that the PS score was higher in cluster B than in cluster A (Supplementary Figure S5). In addition, patients with a high PS-score demonstrated a worse prognosis (Figure 4C). To further evaluate the prognostic capacity of the PS-score in more groups, the robustness of this model was then verified in two other independent GEO-BC groups and the same results were obtained (Figures 4D,E). Univariate and corresponding multivariate Cox regression analyses were further performed and the results demonstrated that the PS-score could serve as an independent prognostic indicator for OS (Figures 4F,G).

Given the significance of the PS-score in predicting the prognosis of BC patients, we next explored its value for clinical applications via integration of the PS-score and clinical characteristics to establish a nomogram to predict the survival rates of GC patients at 3, 5, and 10 years (Figure 4H). Clinical characteristics in this nomograph were considered to exhibit a certain prognostic effect on the clinical outcome of BC and were easily accessible. In this nomogram, each feature was assigned points according to its risk contribution to the overall survival. Meanwhile, the corresponding calibration plot comparing the predicted outcomes with the actual outcome indicated that the nomogram had a certain predictive value (Supplementary Figures S5A, B). The DCA curve was then presented to evaluate the net benefits of our model and the results indicated that compared with a single factor, integration of the clinical stage and the PS-score suggested the optimal clinical efficiency (Figure 4I). These results confirmed that the PS-score plays a crucial role in predicting the prognosis of BC patients.

### Functional Characteristics of Bladder Cancer Associated With the Pyroptosis Score

**FIGURE 5:**
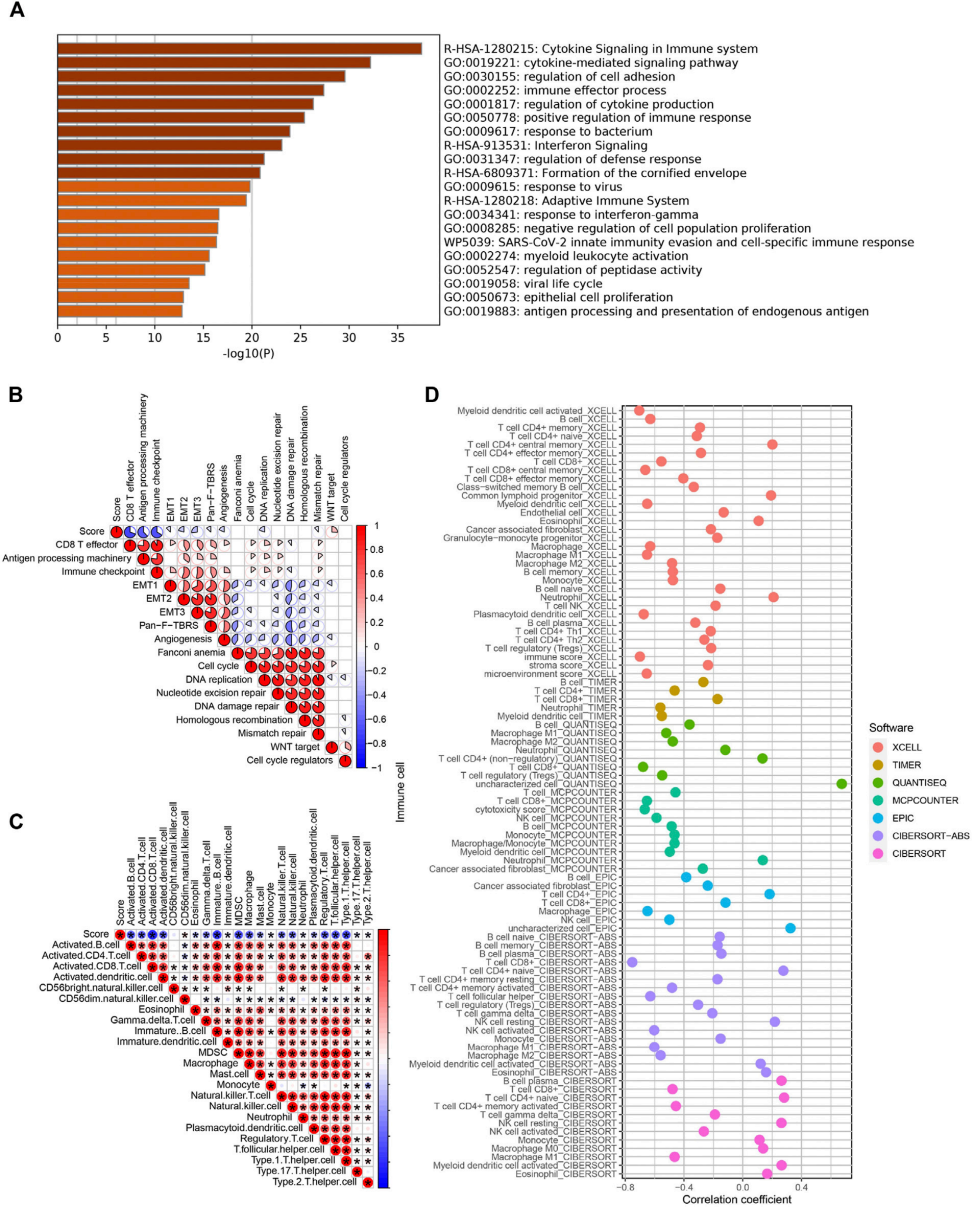
Functional characteristics of BC associated with the PS-score. **(A)** Bar graph of enriched terms for the GO enrichment analysis. **(B)** Correlations between the PS-score and the known biological processes in TCGA cohort. **(C)** Correlation analysis between the PS-score and infiltrating immune cells analyzed by ssGSEA. **(D)** Representative results of the correlation analysis between the PS-score and immune cell infiltration.

We then performed an enrichment analysis to further characterize the features of the pyroptosis-related signatures identified previously. We found that these genes showed enrichment in some biological processes and signaling pathways, particularly those correlated with cytokine signaling, immune effector process, and regulation of immune response (Figure 5A). Moreover, the correlation between the PS-score and known biological pathways demonstrated that the PS-score was negatively correlated with CD8 effector, immune checkpoint, and antigen processing machinery, but positively correlated with WNT targets (Figure 5B). To validate pathway alterations, infiltration of TME cells was further analyzed using the ssGSEA and four other approaches (Figures 5C,D). Surprisingly, the abundance of activated tumor-killing immune cells, including activated CD8^+^ T cells, NK cells, and M1 macrophages, demonstrated a significant negative association with the PS-score. These results indicated that a low PS-score showed an elevated recruitment of effector immune cells and was considered “hot” tumor immune status (Maleki Vareki 2018); a high PS-score was characterized by a poor infiltration by the effector immune cells and high Wnt activation. Therefore, the PS-score might have a predictive value for the response of immunotherapy.

### The Role of Pyroptosis Score and Potential Efficacy of Immunotherapy

**FIGURE 6:**
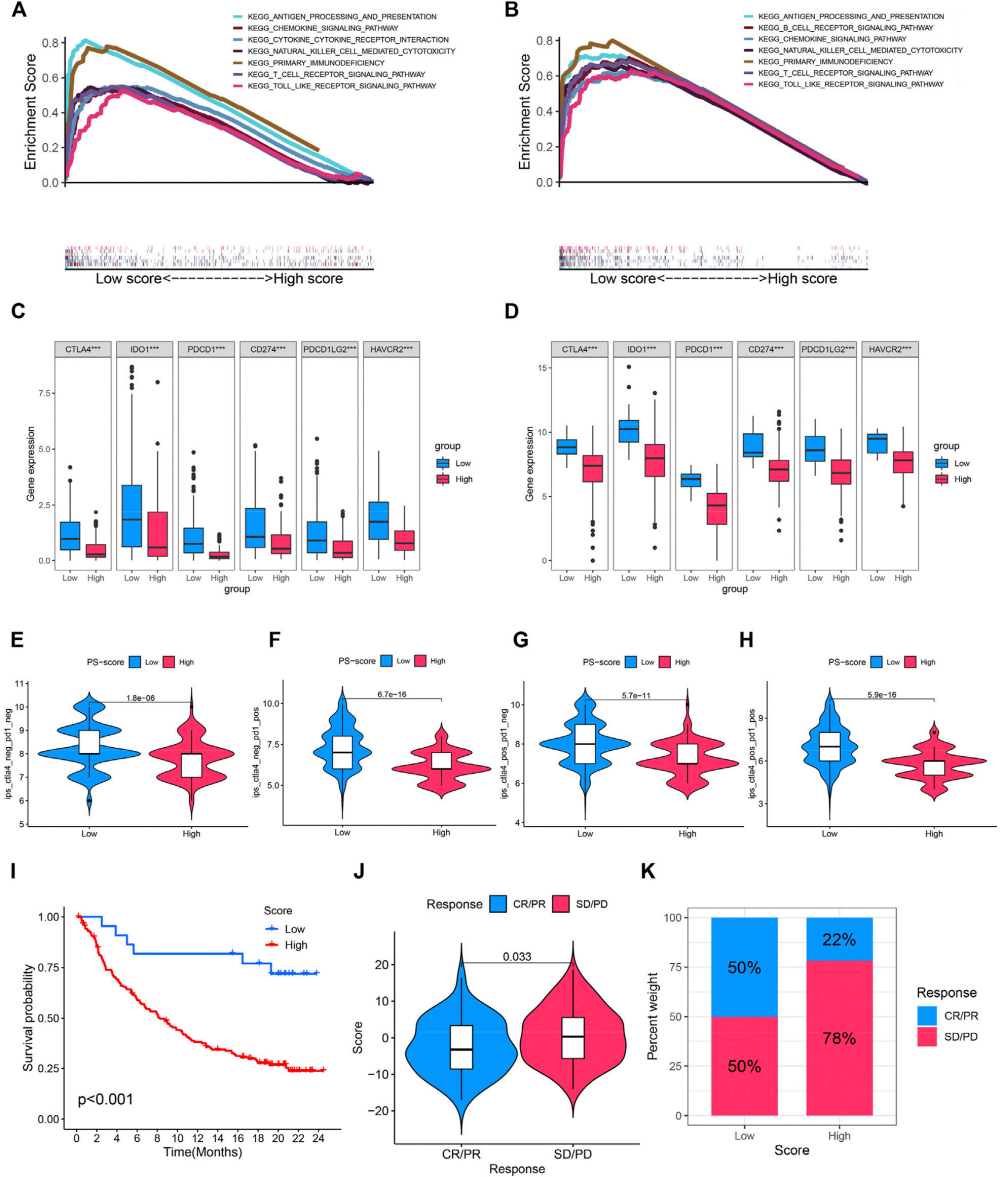
Role of the PS-score and potential efficacy of immunotherapy. **(A,B)** Enriched immune-related gene sets in TCGA and IMvigor210 cohorts with a distinct PS score. **(C,D)** Comparison of immune checkpoint molecules (CD274, PDCD1, IDO1, CTLA4, HAVCR2, and PDCD1LG2) among distinct clusters. **(E–H)** Violin plot showing the association between the IPS and PS score. **(I)** Kaplan–Meier curves for patients with a distinct PS-score in anti-PD-L1 immunotherapy cohort. **(J)** Comparison of the PS scores in different anti-PD-L1 immunotherapy response cohorts. **(K)** Proportion of patients with response to PD-L1 inhibitor treatment in low and high PS-score cohorts. ***p < 0.001.

Immune checkpoint therapy has undoubtedly emerged as a great breakthrough in cancer treatment. To further investigate the potential capability of this model, the GSEA enrichment analysis was carried out in TCGA and IMvigor210 cohorts with anti-PD-L1 immunotherapy. We found pathways associated with immune activation, including antigen presentation and processing, and cytokine signaling , and natural killer cell-mediated cytotoxicity was significantly enriched in BC patients with a low PS-score compared with those with a high PS- score (Figures 6A,B). In addition, immune checkpoint molecules (CD274, PDCD1, IDO1, CTLA4, HAVCR2, and PDCD1LG2) were significantly abundant in the low PS-score group (Figures 6C,D). In addition, the immunogenicity of the two groups was evaluated by the IPS analysis, from which we discovered that the IPS values were upregulated in the low score group (Figures 6E–H). A higher IPS score was previously reported to be positively correlated with increased immunogenicity (Maleki Vareki 2018). In the immunotherapy cohort, the low PS-score group showed an obviously prolonged survival and presented a markedly clinical benefit (Figure 6I). Patients with a lower score exhibited a more partial response (PR) or complete response (CR) (Figure 6J). In addition, we observed a markedly enhanced response to anti-PD-L1 immunotherapy in patients with a low score than those with a high score (50 vs. 22%) (Figure 6K). Thus, the PS-score could be implemented as a potential biomarker for the response of effective immune checkpoint immunotherapy.

### The Association Between Pyroptosis Score and Tumor Mutation Burden

**FIGURE 7:**
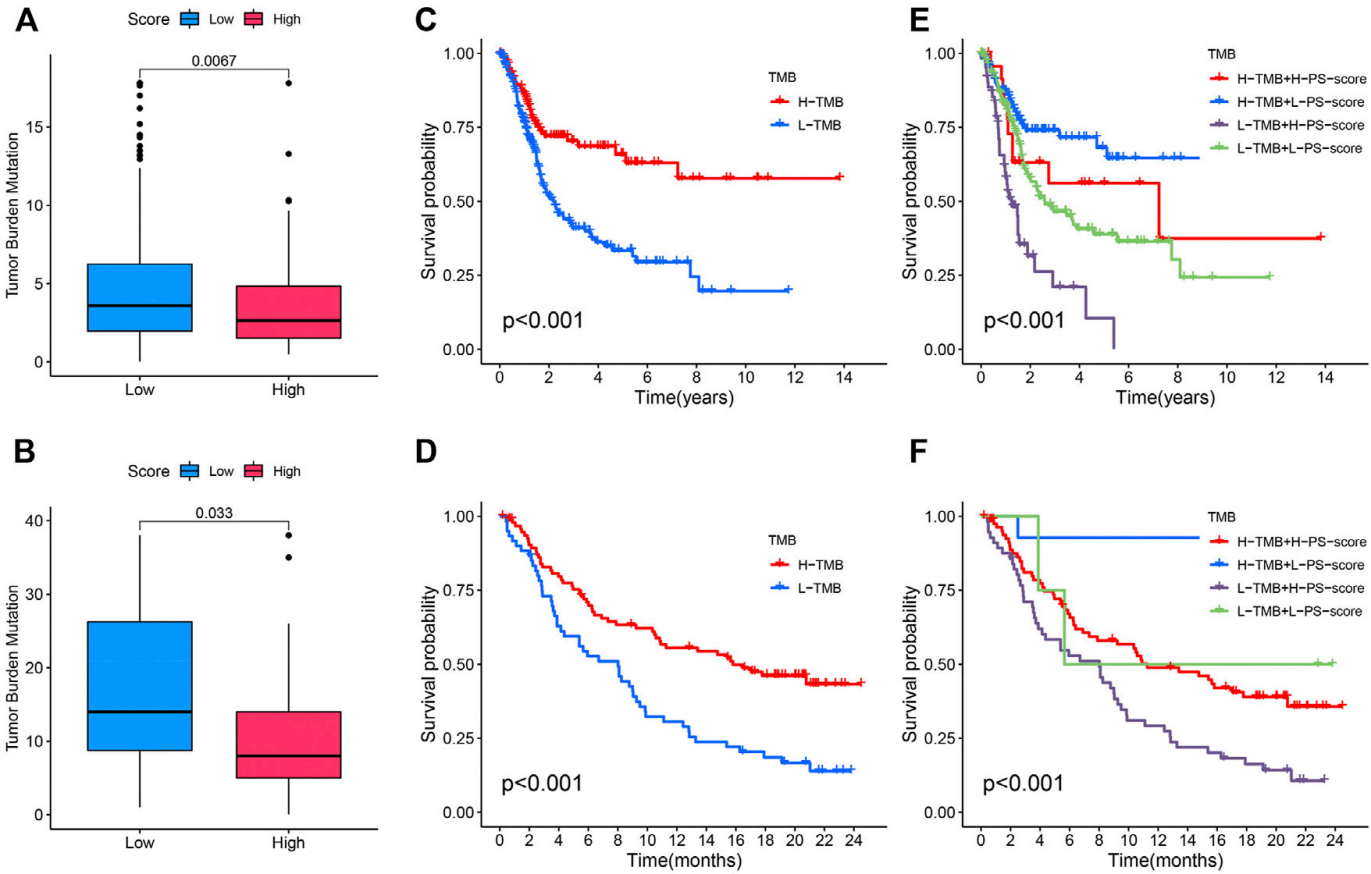
Association between the PS-score and tumor mutation burden (TMB). **(A,B)** Difference of TMB between patients with a distinct PS-score in TCGA and IMvigor210 cohorts. **(C,D)** Kaplan–Meier curves for the distinct groups with high and low TMB levels in the two cohorts. **(E,F)** Kaplan–Meier curves for patients stratified by both the TMB and PS score in the two cohorts.

Studies have revealed that high tumor burden mutation (TMB) was significantly positively correlated with tumor neoantigens, which can be recognized by infiltrating CD8^+^ T cells, thus predicting immunotherapy effects (Rizvi et al., 2015; McGranahan et al., 2016; Chan et al., 2019). Fortunately, in TCGA and IMvigor210 cohorts with anti-PD-L1 immunotherapy, we discovered that a higher TMB was concentrated on patients with a low PS-score (Figures 7A,B). Moreover, ass demonstrated in the Kaplan–Meier analysis, a high TMB suggested a better prognosis (Figures 7C,D). We then explore the cooperative effect of the TMB and PS-score for the prognostic prediction of BC. A stratified survival analysis showed that patients with a higher TMB and high PS-score exhibit the worst outcome (Figures 7E,F). From the aforementioned results, we can conclude that the combined assessment of the TMB and PS-score may be predictive of the response to immune checkpoint inhibitors in advanced BC.

### Functions of the Identified Biomarker in Bladder Cancer Progression

**FIGURE 8:**
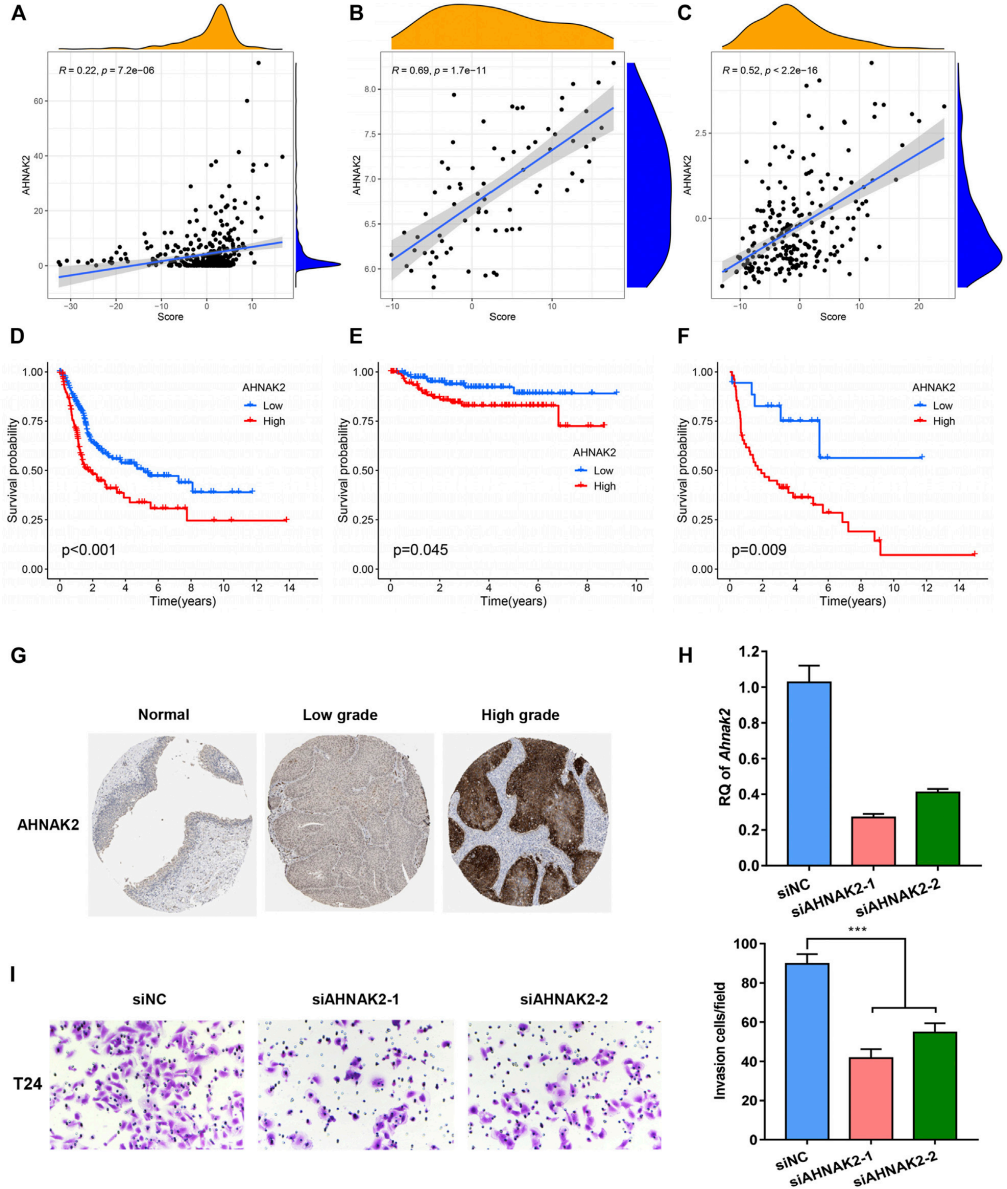
Functions of the identified biomarker in BC progression. **(A-C)** Relationship between the expression level of AHNAK2 and PS-score in TCGA, GSE32894, and GSE48075 cohorts. **(D–F)** Kaplan–Meier curves comparing survival among BC patients with distinct AHNAK2 expression in the three cohorts. **(G)** Immunohistochemical (IHC) analysis of AHNAK2 in bladder urothelium and tumor tissues with different grades of malignancy. **(H)** Knockdown of AHNAK2 was confirmed by quantitative PCR. **(I)** *In vitro* transwell invasion assay for analyzing the effect of AHNAK2 knockdown on cell invasion. Scale bar, 100 μm.

A positive association between Wnt target biological pathways, and the PS-score was identified previously. Among the pyroptosis-related signature genes, AHNAK2 knockdown was previously reported to inhibit the Wnt pathway (Lin et al., 2021). In addition, AHNAK2 expression was reported to be significantly positively correlated with the PS-score in TCGA and two GEO-BC cohorts (Figures 8A–C). As demonstrated in the Kaplan–Meier analysis, the high expression level of AHNAK2 indicated a poor prognosis in BC patients (Figures 8D–F). Immunohistochemistry for AHNAK2 showed the positive staining intensity in bladder tumor samples, stronger than in the normal urothelium tissues. Moreover, tumor samples with a high pathological grade revealed stronger expression than tissues with a low grade by HPA (Figure 8G). We then performed functional studies with specific small interfering RNAs (siRNAs) and the results showed that the knockdown of AHNAK2 could inhibit the cell invasion ability (Figures 8H,I). These results confirmed that AHNAK2 was associated with tumor progression, thus affecting prognosis.

## Discussion

BC is among the most prevalent malignancies and exerts an enormous burden on global healthcare. The high probability of the recurrence rate and poor outcome remains a major challenge in the treatment of bladder cancer, especially its muscle-invasive form (Lobo et al., 2017). Optimizing therapeutic options and identifying key biomarkers for patients with advanced BC may contribute to the treatment landscape.

An increasing amount of evidence reveals the indispensable role of pyroptosis in tumorigenesis and anti-tumor activity (Yu et al., 2019; Zeng et al., 2019; Hou et al., 2020). While most studies were dedicated to explore a single molecule, the functions and mechanisms of these molecules remain far from satisfactory. Presently, many studies have identified models based on expression profiles to improve the potential value of personalized options for BC (Yan et al., 2020b; Yan et al., 2021). In this study, we depicted the landscape of these 33 pyroptosis genes at the transcriptional and genetic levels and their correlations in BC. Based on the aforementioned molecules, we defined two pyroptosis subtypes with significantly distinct clinical characteristics, somatic copy number variations, and tumor immune landscape. Most pyroptosis-related molecules exhibited high expression levels in cluster A. Moreover, cluster A indicated a better prognosis and was significantly enriched in apoptosis and immune activation pathways. Activated immune cells, especially tumor-infiltrating lymphocytes such as CD4^+^ cells, CD8^+^ T cells, and NK cells were abundant in cluster A. In addition, immunomodulators, which are essential for cancer immunotherapy, showed significant disparities (Yang 2015; Tang et al., 2018). Studies have demonstrated that GSDMC was positively correlated with PD-L1 expression (Hou et al., 2020). PD-L1 could interact with p-stat3 followed by binding to the GSDMC promoter region, ultimately upregulating GSDMC expression and inducing pyroptosis. These results demonstrated that pyroptosis-related modification could regulate the tumor immune microenvironment.

Considering the high heterogeneity of pyroptosis patterns in individual BC patients, classifying the expression levels of pyroptosis regulatory genes is crucial. We thus established a scoring model, the PS-score, to evaluate the efficiency of pyroptosis patternS in individual BC patients. We confirmed that the PS-score serves as an independent prognostic factor. The PS-score was negatively associated with CD8 effector, antigen-processing machinery, and immune checkpoint biological pathways. There is a negative correlation between the score and the infiltration of anti-tumor TILs. In addition, the negative correlation between the PS-score and immune checkpoint genes suggested that the score model may predict the immunotherapy response. Immunotherapy, such as PD-L1 blockade therapy, has contributed to the curative effectiveness in many advanced malignancies, including advanced BC, while the heterogeneity of the response rate still limits its clinical application (Carosella et al., 2015; Oh et al., 2020). Therefore, identifying biomarkers that can predict and benefit from immunotherapy is urgently required. Surprisingly, in a cohort with anti-PD-L1 immunotherapy, we found that patients with a low PS-score exhibited a better clinical response and a prolonged survival time compared with patients with high scores. The tumor mutational burden (TMB) is also previously reported to be correlated with anti-PD-1 therapy in multiple cancers, including urothelial carcinoma (Bellmunt et al., 2017) and metastatic NSCLC (Hellmann et al., 2019). Consistent with the present study, a high TMB is associated with improved efficacy and better outcomes in BC cohorts treated with anti-PD-L1 immunotherapy. A combined analysis of the TMB and PS-score also indicates that the efficacy of immunotherapy is enhanced in patients with a high TMB and low PS-score compared with those other groups.

Except for the tumor microenvironment, we found that the PS-score was positively correlated with Wnt target biological pathways. Wnt signaling is an essential biological process, which plays critical roles in tumorigenesis and prognosis (Reya and Clevers 2005). Among the DEGs between distinct pyroptosis-related subtypes, AHNAK2 knockdown was previously reported to activate the Wnt pathway and correlated with tumor immune cell infiltration while its role in BC is rarely reported (Lin et al., 2021; Zheng et al., 2021). We found a significant positive association between AHNAK2 expression and the PS-score. A survival analysis suggested that AHNAK2 overexpression predicts poor overall survival and tumor malignancy. In addition, the knockdown of AHNAK2 significantly weakened the invasive capacities of bladder tumor cells. Nevertheless, the possible mechanism that contributes to the carcinogenesis of BC remains to be investigated.

However, we recognize several inevitable limitations in our study. This score signature is a retrospective study and highly context-dependent. In addition, the tumor immune microenvironment was obtained based on algorithms rather than clinical validation. Another main limitation in the present research is the lack of proteomics/metabolomics data sets except for transcriptome data, making it difficult to validate its clinical utility in other omics data. Therefore, large-scale clinical research and protein sequencing further merit exploration in the future.

In summary, this work indicated the correlation between pyroptosis-related patterns and tumor immune remodeling in BC. Our comprehensive assessment of pyroptosis-related patterns in individual BC cases may contribute to the evaluation of the tumor immune landscape and identifying its therapeutic utility in immunotherapy. We hope our research could promote the development of new therapeutic strategies and new immunotherapeutic agents.

## Data Availability

The datasets presented in this study can be found in online repositories. The names of the repository/repositories and accession number(s) can be found in the article/Supplementary Material.
