# Supplementary material for: Pyroptosis-Related Patterns Predict Tumor Immune Landscape and Immunotherapy Response in Bladder Cancer
Source: Front Mol Biosci. 2022 Apr 26;9:815290. doi: 10.3389/fmolb.2022.815290 (PMC9086408; doi:10.3389/fmolb.2022.815290)
Supplement: Supplementary file 1 [file DataSheet1.docx]

Supplementary Material


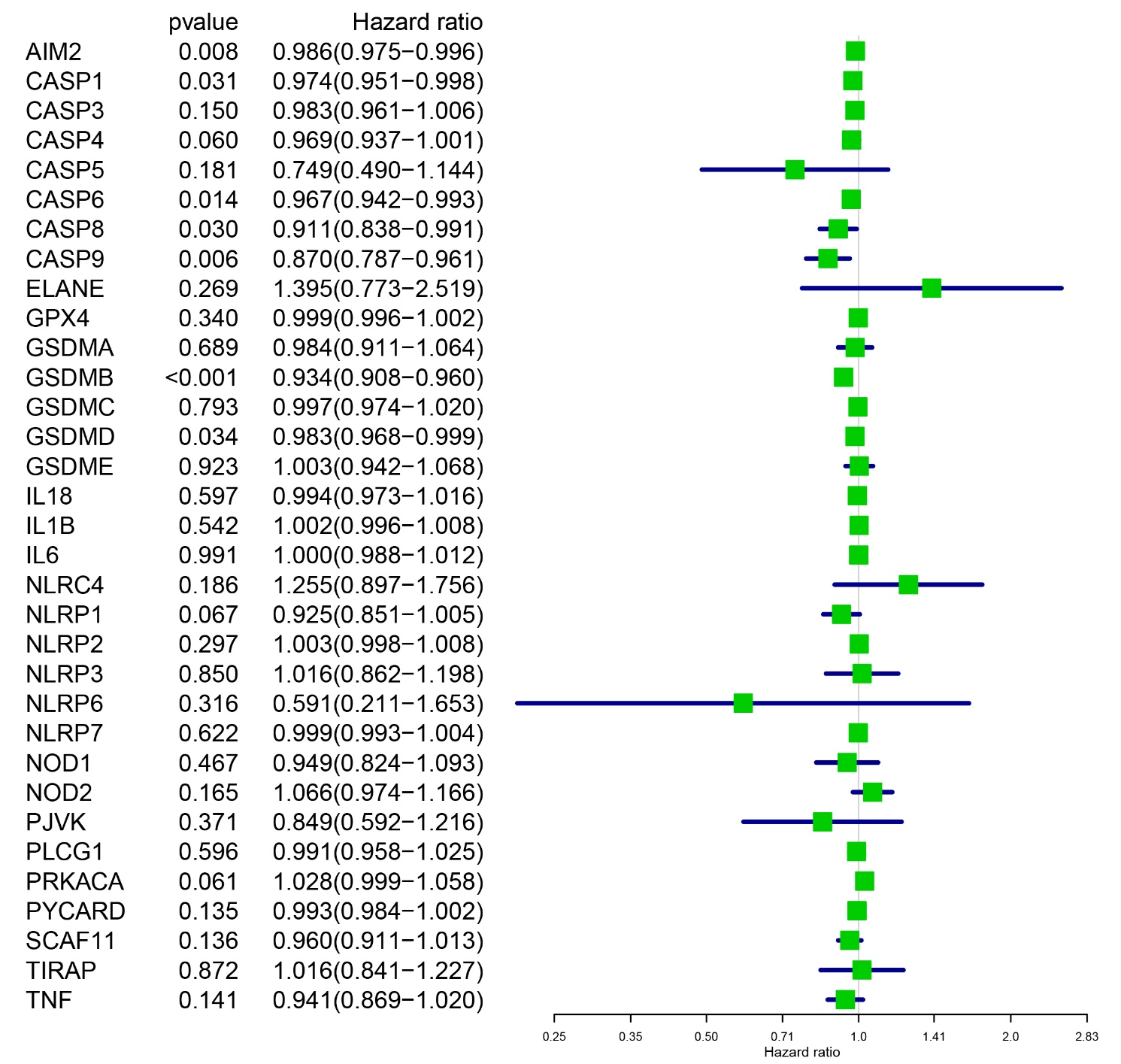


**Supplementary Figure 1.** A simple forest plot of hazard ratio for each gene of 33 PRGs.

**
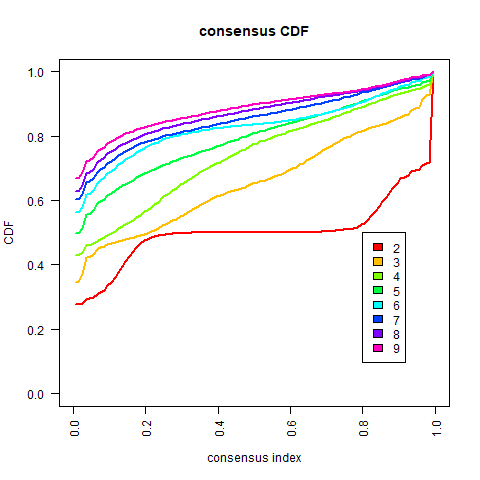
**

**Supplementary Figure 2.** Consensus CDF plot and consensus index for k =2 to 9 are represented. X axis represents consensus index, Y axis represents CDF.


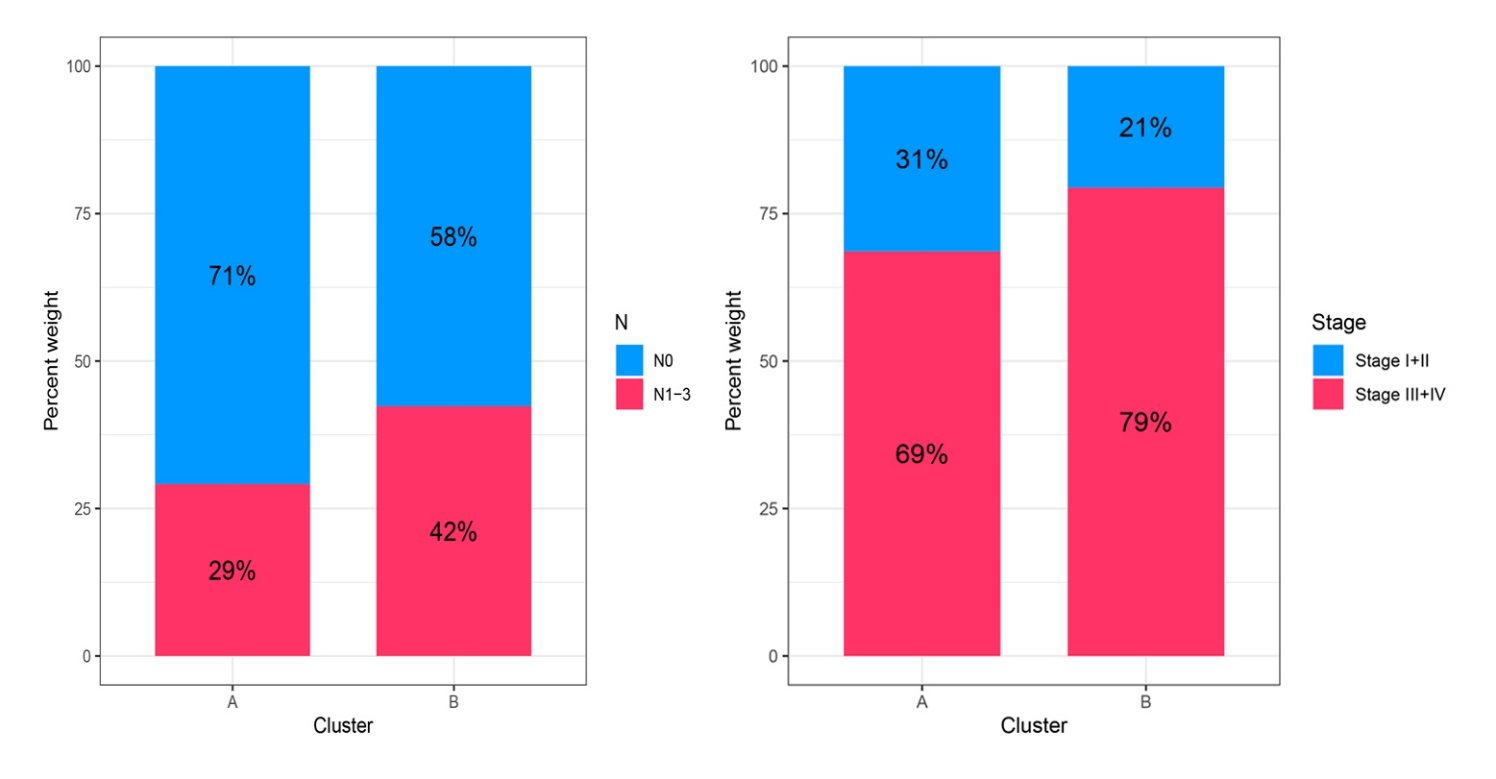


**Supplementary Figure 3.** The proportion of patients with N and stage in cluster A and B cohorts.


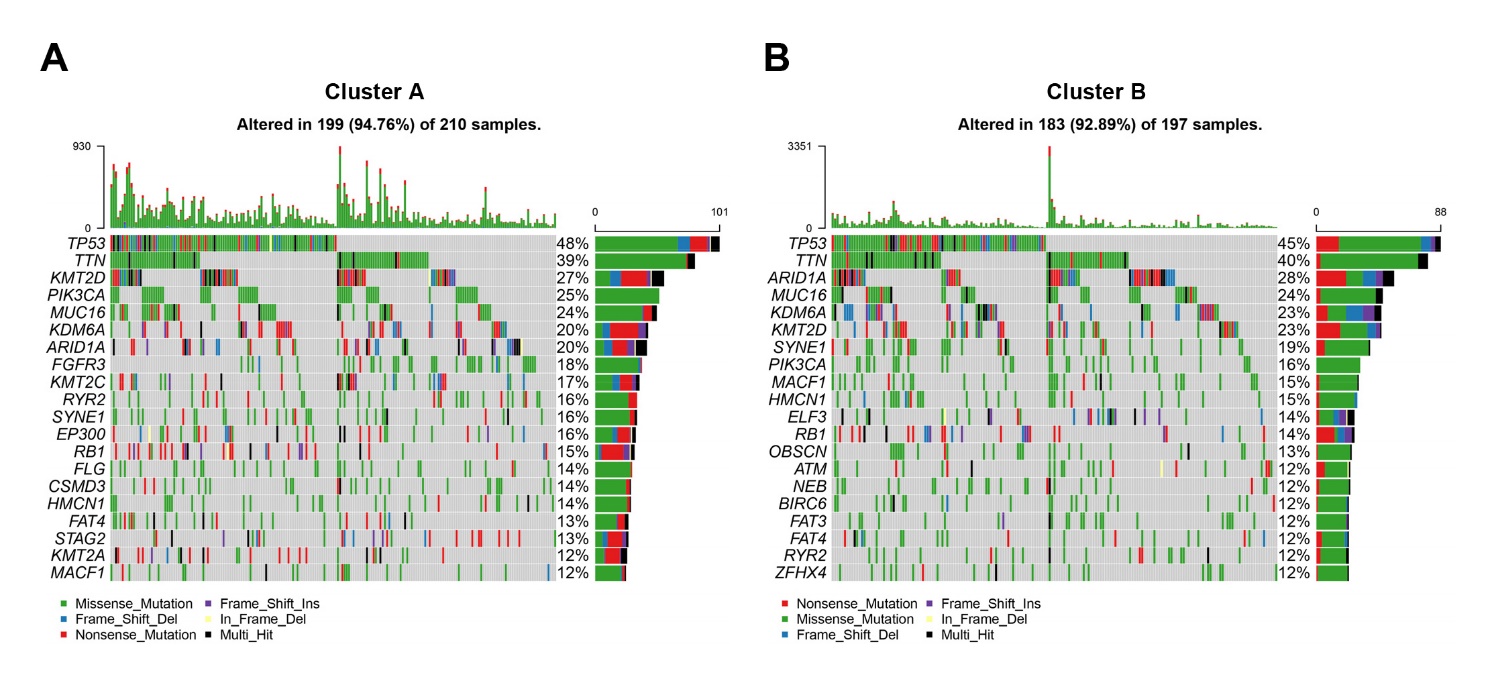


**Supplementary Figure 4.** Landscape of mutation profile of BC patients in distinct pyroptosis-related clusters.


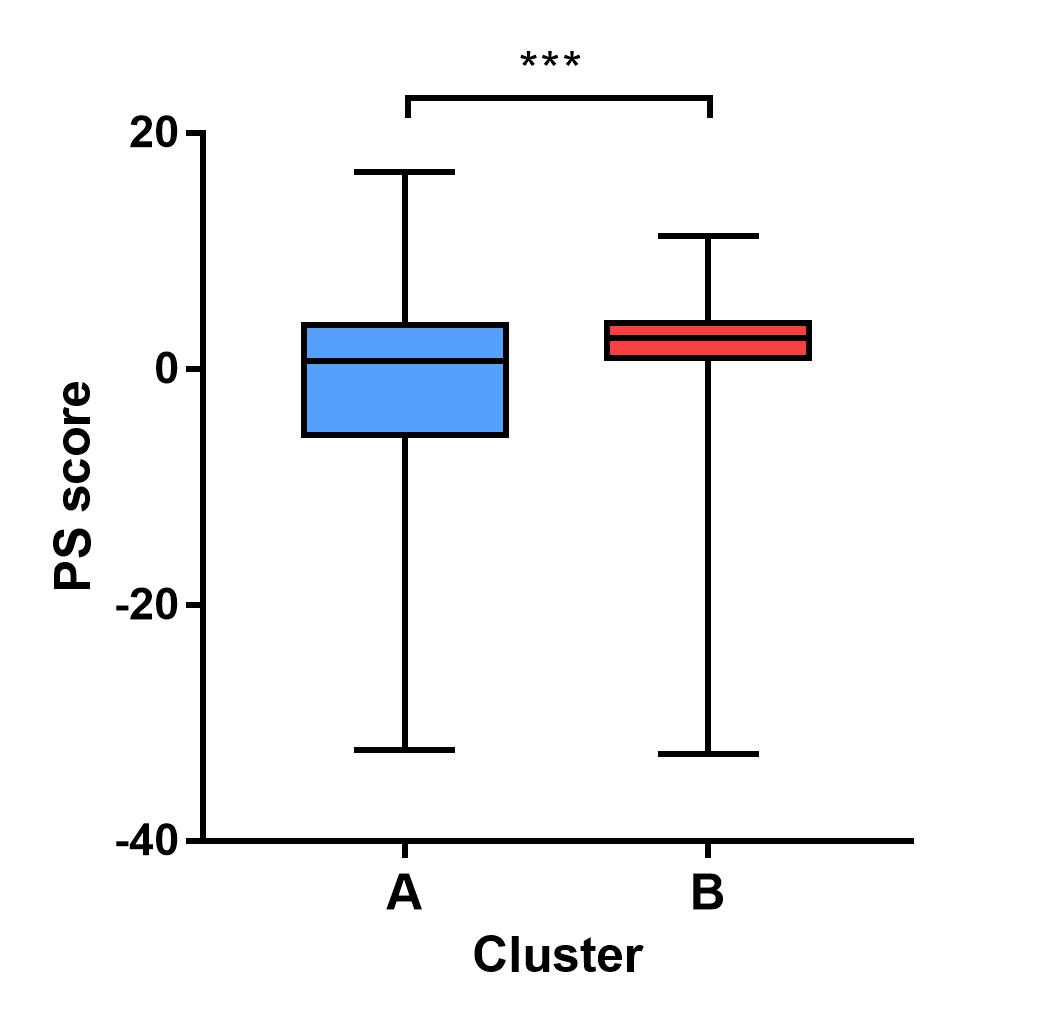


**Supplementary Figure 5.** The Kruskal–Wallis test shows the PS score at two different levels of pyroptosis. The line in the box represents the median.


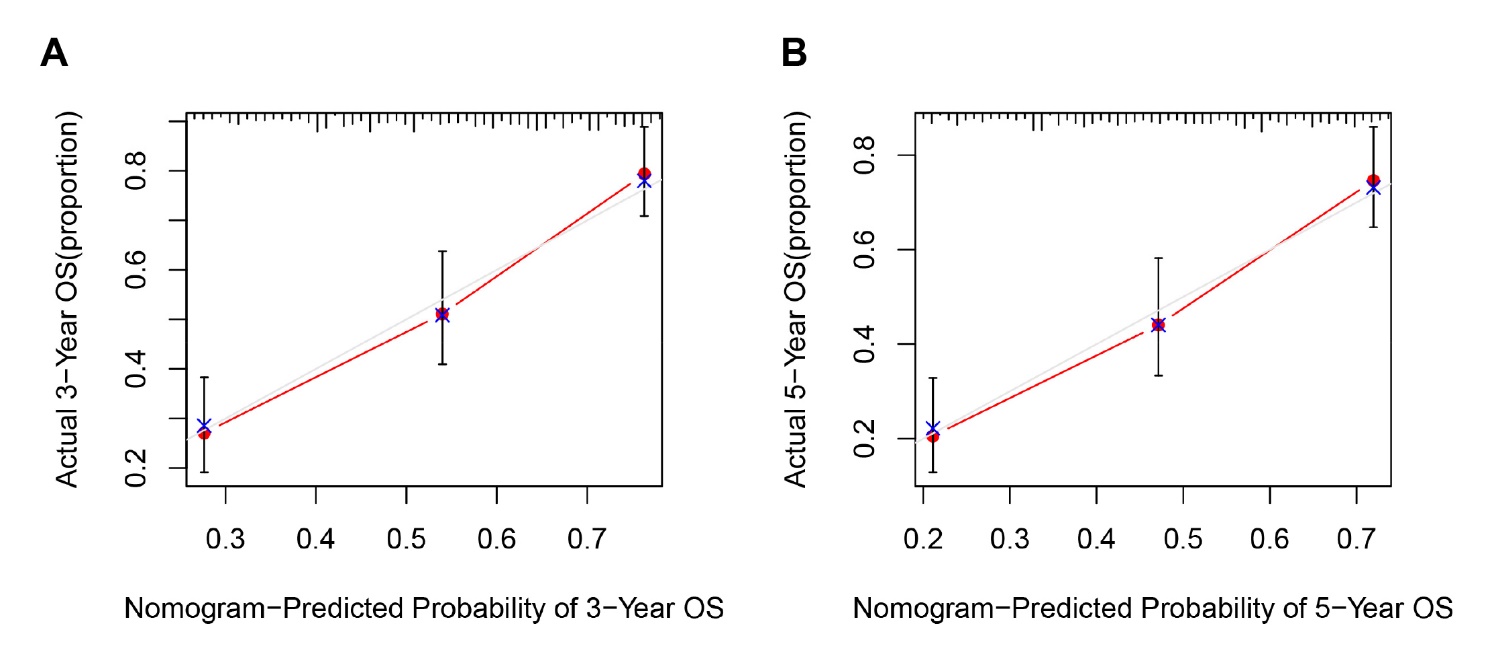


**Supplementary Figure 6.** Calibration curves of the nomogram between predicted and observed 3-year and 5-year outcomes.
